# Inflammatory Microenvironment and Specific T Cells in Myeloproliferative Neoplasms: Immunopathogenesis and Novel Immunotherapies

**DOI:** 10.3390/ijms22041906

**Published:** 2021-02-14

**Authors:** Vincenzo Nasillo, Giovanni Riva, Ambra Paolini, Fabio Forghieri, Luca Roncati, Beatrice Lusenti, Monica Maccaferri, Andrea Messerotti, Valeria Pioli, Andrea Gilioli, Francesca Bettelli, Davide Giusti, Patrizia Barozzi, Ivana Lagreca, Rossana Maffei, Roberto Marasca, Leonardo Potenza, Patrizia Comoli, Rossella Manfredini, Antonino Maiorana, Enrico Tagliafico, Mario Luppi, Tommaso Trenti

**Affiliations:** 1 Department of Laboratory Medicine and Pathology, Diagnostic Hematology and Clinical Genomics, AUSL/AOU Policlinico, 41124 Modena, Italy; g.riva@ausl.mo.it (G.R.); beatrice.lusenti@gmail.com (B.L.); enrico.tagliafico@unimore.it (E.T.); t.trenti@ausl.mo.it (T.T.); 2 Section of Hematology, Department of Medical and Surgical Sciences, University of Modena and Reggio Emilia, AOU Policlinico, 41124 Modena, Italy; paolini.ambra@aou.mo.it (A.P.); fabio.forghieri@unimore.it (F.F.); maccaferri.monica@aou.mo.it (M.M.); messerotti.andrea@aou.mo.it (A.M.); pioli.valeria@aou.mo.it (V.P.); gilioli.andrea@aou.mo.it (A.G.); francesca.bettelli@unimore.it (F.B.); davide.giusti@unimore.it (D.G.); patrizia.barozzi@unimore.it (P.B.); ivana.lagreca@unimore.it (I.L.); rossana.maffei@unimore.it (R.M.); roberto.marasca@unimore.it (R.M.); leonardo.potenza@unimore.it (L.P.); mario.luppi@unimore.it (M.L.); 3 Institute of Pathology, Department of Medical and Surgical Sciences, University of Modena and Reggio Emilia, AOU Policlinico, 41124 Modena, Italy; roncati.luca@aou.mo.it (L.R.); antonino.maiorana@unimore.it (A.M.); 4 Pediatric Hematology/Oncology Unit and Cell Factory, Istituto di Ricovero e Cura a Carattere Scientifico (IRCCS) Policlinico San Matteo, 27100 Pavia, Italy; pcomoli@smatteo.pv.it; 5 Centre for Regenerative Medicine “S. Ferrari”, University of Modena and Reggio Emilia, 41125 Modena, Italy; rossella.manfredini@unimore.it

**Keywords:** MPN, inflammation, immunity, T cells, niche, PV, ET, PMF, JAK2, CALR

## Abstract

The Philadelphia-negative myeloproliferative neoplasms (MPNs) are malignancies of the hematopoietic stem cell (HSC) arising as a consequence of clonal proliferation driven by somatically acquired driver mutations in discrete genes (JAK2, CALR, MPL). In recent years, along with the advances in molecular characterization, the role of immune dysregulation has been achieving increasing relevance in the pathogenesis and evolution of MPNs. In particular, a growing number of studies have shown that MPNs are often associated with detrimental cytokine milieu, expansion of the monocyte/macrophage compartment and myeloid-derived suppressor cells, as well as altered functions of T cells, dendritic cells and NK cells. Moreover, akin to solid tumors and other hematological malignancies, MPNs are able to evade T cell immune surveillance by engaging the PD-1/PD-L1 axis, whose pharmacological blockade with checkpoint inhibitors can successfully restore effective antitumor responses. A further interesting cue is provided by the recent discovery of the high immunogenic potential of JAK2V617F and CALR exon 9 mutations, that could be harnessed as intriguing targets for innovative adoptive immunotherapies. This review focuses on the recent insights in the immunological dysfunctions contributing to the pathogenesis of MPNs and outlines the potential impact of related immunotherapeutic approaches.

## 1. Introduction

The Philadelphia-negative myeloproliferative neoplasms (MPNs) are clonal disorders of the hematopoietic stem cell (HSC) characterized by the proliferation of mature myeloid cells in the bone marrow (BM). MPNs are traditionally classified on the basis of the BM morphology and the amount of fibrosis, combined with clinical, laboratory, cytogenetic and molecular features. The three most common entities are essential thrombocythemia (ET), polycythemia vera (PV) and primary myelofibrosis (PMF). ET is chiefly characterized by megakaryocyte expansion, accounting for an increased platelet count, whereas patients with PV typically display an elevated red cell mass, usually observed along with leukocytosis and thrombocytosis, consistently with trilineage proliferation (panmyelosis) detected in the BM. The distinguishing features of PMF are peripheral leuko-erythroblastosis, massive splenomegaly and BM fibrosis [1].

The incidences of ET and PV are quite similar, at 1–2 cases per 100,000 person-years in the United States, while PMF is less frequent, with an incidence of 0.3 per 100,000 person-years [2]. Although this aberrant overproduction of functional mature cells may initially present as an indolent disorder, with patients (especially those with ET or PV) displaying only asymptomatic peripheral cytoses, all MPNs harbor a potential for evolution, either to an end-stage myelofibrosis (MF) with BM failure or to overt acute leukemia [3]. In addition, the clinical course of MPNs is characteristically complicated by thrombotic and bleeding events [4]. As a consequence, MPN cases globally have a lower life expectancy and a worse quality of life compared to the healthy population [5]. The evolutive risk significantly varies among nosologic entities, being influenced by several factors, with driver and non-driver mutations acting as master determinants [6].

The mutational landscape of MPNs is rather homogeneous, as these neoplasms share a high incidence of the acquired point mutation (V617F) in the gene encoding Janus kinase 2 (JAK2), which is observed in approximately 98% of patients with PV and 50–60% of patients with PMF and ET [7]. The JAK2V617 mutation induces a loss of function of the JH2 pseudo-kinase domain of JAK2, which switches JAK2 to a constitutively active state, leading to an aberrant engagement of downstream signaling pathways, such as signal transducer and activator of transcription 3 (STAT3), STAT5, mitogen-activated protein kinases (MAPKs) and the protein kinase B pathway (PI3K/Akt), followed by increased cell proliferation and systemic hyper-inflammation [8]. In 2007, additional JAK2 mutations (in exon 12) were described in JAK2V617F-negative patients with PV [9]. The understanding of the molecular pathogenesis of MPNs was further improved by the discovery of the mutually exclusive somatic mutations of myeloproliferative leukemia protein (MPL; thrombopoietin receptor, TPOR) and calreticulin (CALR) genes in patients with wild-type JAK2 ET or PMF [10,11,12]. Gain-of-function MPL mutations, causing a substitution of tryptophan at position W515 by leucine (MPLW515L) or lysine (MPLW515K), constitutively activate downstream signaling in a ligand-independent manner [13]. Mutations of CALR, a chaperone multifunctional calcium-binding protein localized in the endoplasmic reticulum, consist of deletions and/or insertions in exon 9, resulting in a novel positively charged amino acid sequence in the C-terminus [14]. More than 50 CALR mutations have been reported to date, with only two types (a 52-bp deletion and a 5-bp insertion) accounting for approximately 80% of cases. Although the oncogenic mechanism of the mutant CALR is not fully elucidated, it has been hypothesized that the mutations enhance the binding capacity of CALR to TPOR, whose hyperactivation leads to enhanced megakaryocyte proliferation and thrombocytosis [15,16]. All these clonal markers are formally integrated into the WHO diagnostic criteria for PV (98% JAK2 mutational frequency), ET (60% JAK2, 22% CALR and 3% MPL) and PMF (58% JAK2, 25% CALR and 7% MPL); about 10–15% of patients with PMF or ET lack all the three driver mutations and are generally referred to as “triple negative” [7,17]. Almost one third of the patients with MPNs harbor non-driver molecular lesions that might cooperate with the driver mutations in fostering disease progression [18,19]. The genes involved are those relevant to epigenetic (e.g., TET2, ASXL1, IDH1, IDH2, DNMT3A, EZH2), RNA splicing (e.g., SF3B1, SRSF2, U2AF1) or transcriptional (e.g., IKZF1, TP53, NF-E2, CUX1) regulation, some of which carry prognostic information, especially in PMF [7,20,21,22]. Interestingly, it has been reported that the sequential induction of DNMT3A and nucleophosmin (NPM1) mutations in genetically engineered mice can generate an MPN-like disorder, following a condition of clonal hematopoiesis [23]. Nonetheless, NPM1 mutations are not found in MPNs in humans and are only exceptionally detected in non-acute myeloid neoplasms sharing dysplastic features (either purely myelodysplastic or myelodysplastic/myeloproliferative), multilineage involvement and excess of blasts, causing controversies about the pathological classification of these uncommon cases [24,25,26].

Recently, in parallel with the advances in molecular characterization, there is increasing evidence that inflammation plays a crucial role in promoting MPN initiation and affecting disease evolution. Moreover, several studies demonstrated that the immune system is profoundly disrupted in MPNs, so as to enable mechanisms of tumor escape. Therefore, it is conceivable that, further to the pharmacological inhibition of JAK-STAT, the recovery of protective specific tumor immune surveillance could be potentially exploited for therapeutic purposes.

In this review article, we discuss the mechanisms underlying the immune dysregulation in MPNs and summarize the related novel immunotherapeutic approaches with promising disease-modifying potential.

## 2. Hit the Road JAK: JAK-STAT Signaling at the Dangerous Crossroads between Inflammation and Clonal Myeloproliferation

Chronic inflammation and oxidative stress have been considered hallmarks of MPNs for decades. The discovery of the activating mutations of JAK2 has provided a biological rationale for this tight link, as Janus kinases mediate the signal transduction of cytokines, chemokines and growth factors, and constitute pivotal pathways in the immune system, being able to promote proliferation, differentiation and cytokine production in HSCs and progenitor cells, as well as mature immune cells [27]. The JAK-STAT signaling cascade can be pathologically hyperactivated either by excessive ligand binding (such as in the case of chronic inflammatory conditions) or by activating mutations in gene loci encoding Janus kinases (as observed in MPNs) [28].

The pathogenetic role of inflammation has been emphasized in the initiation and progression of disease, as well as in the development of the typical signs/symptoms of MPNs, such as anemia, constitutional symptoms, thrombosis, splenomegaly, BM fibrosis and pulmonary hypertension, thus actively contributing to morbidity and mortality [29,30,31,32]. Moreover, for some authors, MPNs epitomize a full-fledged inflammatory model of human cancer development, as suggested by the evidence of abnormal cytokine production and an association with several inflammatory/autoimmune diseases and second cancers [33,34,35,36,37,38]. In this model, chronic inflammation is hypothesized to induce the first oncogenic hit in the HSC by causing mutations and genomic instability, eventually leading to MPN emergence [39,40]. Alternatively, mutations (exemplified by JAK2V617F) can randomly occur in the aged BM. Oncogenic lesions constitutively activate inflammatory pathways in HSCs and progenitor cells, eliciting the production of reactive oxygen species (ROS) and pro-inflammatory cytokines [41,42]. At early stage, the disease may be not clinically apparent and could be described as clonal hematopoiesis with a sub-clinical inflammatory state. The accumulation of ROS in mutated cells damages DNA and favors clonal proliferation, driving disease progression towards full-blown MPN [43,44,45]. In particular, JAK2V617F mutation leads to the upregulation of plenty of cytokines, chemokines and growth factors, including interleukins IL-1β, IL-6, IL-8, IL-10, IL-11, IL-12, IL-15, IL-17 and IL-33, chemokine (C-X-C motif) ligand 1 (CXCL1), CXCL4, tumor necrosis factor-α (TNF-α), transforming growth factor-β (TGF-β), granulocyte macrophage-colony stimulating factor (GM-CSF), platelet-derived growth factor (PDGF), vascular endothelial growth factor (VEGF) and angiopoietin-1, as reported in murine models and in patients’ samples [46,47,48,49,50,51,52]. Most of the abovementioned molecules are either pro-inflammatory or directly pro-fibrotic (with the exception of IL-10 which has an anti-inflammatory function), and hold prognostic significance, being associated with clinically relevant outcomes [53,54]. By way of example, TGF-β acts as a potent inducer of fibrosis [55], while IL-8 has been linked with the presence of constitutional symptoms and leukemic transformation of PMF [53,56]. Likewise, in the setting of PV, levels of IL-12, IL-1β and interferon (IFN, either α or γ) correlate with hematocrit values, leukocytosis and risk of thrombosis, respectively [57]. The activation of the nuclear factor kappa B (NF-κB) and hypoxia-inducible factor-α (HIF-α) signaling pathways further enhances the production of these soluble factors, giving rise to a vicious cycle that is continuously fueled by the release of inflammatory mediators from activated neutrophils, monocytes and platelets [58,59]. Consistent with this, leukocytosis constitutes an independent risk factor for thrombosis. In more detail, different JAK2-induced pathophysiologic mechanisms contribute to the typically high thrombotic risk of MPNs: (i) blood viscosity related to hyper-cellularity and (ii) alterations in (a) plasmatic coagulation (b) vessel walls, (c) cell adhesion and function [4].

Notably, monocytosis is an independent unfavorable prognostic factor for overall survival in patients with PV and PMF [60,61]. Monocytes, whether classical (CD14+ CD16−), non-classical (CD14− CD16+) or intermediate (CD14+ CD16+), represent key cellular mediators of inflammation, thrombosis and BM fibrosis, contributing to MPN pathogenesis through multifaceted mechanisms. As highlighted by several studies, MPN-associated monocytes, regardless of their mutational status, (i) secrete large amounts of cytokines, such as TNF-α, TGF-β and IL-10 [62], (ii) show impaired response to anti-inflammatory IL-10 (often elevated in MPNs) [63], (iii) stimulate osteoclastogenesis within the endosteal niche [64] and (iv) give origin to a population of clonal monocyte-derived fibrocytes, co-expressing markers of hematopoietic cells (e.g., CD34, CD45 and CD68) and stromal cells (e.g., collagen I, collagen III and fibronectin) involved in the induction of BM fibrosis in PMF [65]. Furthermore, CD56+CD14+ pro-inflammatory monocytes have recently been identified in ET as a source of increased CXCL1 levels, which correlate with evolution towards MF [66].

Since Janus kinases work as downstream signal transmitters of many cytokine receptors, JAK2 mutations have been more clearly linked to inflammatory processes. Nevertheless, akin to JAK2V617F, somatic mutations of MPL and CALR, as well as age-related gene mutations involved in clonal hematopoiesis of indeterminate potential (CHIP), are also known to exhibit inflammatory signatures [59,67,68]. As one prominent example, loss-of-function TET2 mutations have been described to be associated with increased levels of IL-1β, IL-6 and IL-18 [69,70,71]. However, most upregulated inflammatory pathways converge on the JAK/STAT signal transduction axis, being dependent on JAK activity [27].

## 3. Tainted Neighborhood: The Emerging Role of the Bone Marrow Niche

The BM niche is the highly specialized microenvironment where HSCs reside and flourish. This complex structure, whose role is essential for HSC functional preservation, includes both cellular and non-cellular components, and can be schematically divided into three compartments: the endosteal niche, the perivascular niche and the extracellular matrix (ECM). The endosteal niche is mainly formed by osteoblasts, osteoclasts, osteocytes and a peculiar osteoblast-derived population termed spindle-shaped N-caderin+ osteoblasts (SNO cells). The perivascular niche contains sinusoidal endothelium, arterioles, transition zone vessels, mesenchymal stem cells (MSCs) and CXCL12-abundant reticular cells. The ECM is a non-cellular space that provides integrity for the niche, as well as acting as an HSC regulator. The finely orchestrated “cross-talk” among the aforementioned players allows HSC survival, as well as their adaptation to external stimuli (such as inflammation), thus driving HSC fate [72]. In brief, under the steady state, several factors, such as CXCL12 or KIT ligand (released by diverse MSC populations), Jagged-1 or developmental endothelial locus-1 (DEL-1) (produced by endothelial cells), CXCL4 and TGF-β1 (secreted by megakaryocytes), cooperate in the maintenance of HSCs, which mostly dwell in the proximity of BM vessels (arterioles or sinusoids). Upon infection or other inflammatory stimuli, niche populations modulate the hematopoietic response by enhancing the release of granulocyte colony-stimulating factor (G-CSF) and IL-6, in order to promote the proliferation and differentiation of myeloid progenitors (emergency myelopoiesis), whereas the expression of CXCL12 and KIT ligand is downregulated. IL-6 is secreted either by MSCs (especially after stimulation with IFN-γ produced by cytotoxic T cells) or endothelial cells; the latter represents the main source of G-CSF as well [73].

Although many dynamics of the BM microenvironment, both in physiological and pathological states, still remain to be established, a growing number of studies indicate that MPN-associated BM niche homeostasis is disrupted at multiple levels, contributing to the proliferation, survival and migration of mutated HSCs [74]. It has been reported that JAK2V617F+ long-term HSCs (LT-HSCs) are able to initiate the disease, since this mutation confers a clonal advantage to dominate the niche over wild-type LT-HSCs, whose repopulation capacity is conversely hindered by elevated JAK2 expression levels [75,76]. Finally, the acquisition of a second mutational hit (such as TET2 mutations) provides a further clonal advantage, favoring disease progression [77]. Alternatively, a mutation of TET2 may precede JAK2V617F, accounting for different clonal hierarchies and clinical phenotypes, which were comprehensively reviewed elsewhere by Mead and Mullally [78]. Besides the clonal architecture of the stem cell pool, an aberrant functionality of both endosteal and perivascular niches, as well as the ECM, plays a critical role in the pathogenesis of MPNs.

As regards the endosteal niche, in a murine model of MPN, Schepers et al. demonstrated an abnormal osteoblast expansion due to overstimulation by MSCs, associated with the overproduction of inflammatory cytokines, the promotion of fibrogenesis and the downregulation of CXCL12 expression (which is essential for the maintenance of quiescent HSCs and controlled HSC mobilization), leading to the establishment of a “self-reinforcing” MPN niche [79]. Moreover, recent studies in the setting of JAK2V617F+ MPNs showed that clonally derived monocytes can stimulate osteoclastogenesis, generating an osteoclast-enriched microenvironment, which further ensures the survival and expansion of MPN cell populations over normal hematopoiesis [64].

Mutant HSCs can also perturb the well-balanced vascular niche by upregulating several targets involved in neo-angiogenesis (e.g., VEGF and angiopoietin-1) and fibrogenesis (e.g., TGF-β1, CXCL4, PDGF, IL-1β and TNF-α), in order to establish a permissive and nourishing milieu [80,81,82,83]. In particular, TGF-β1, whose levels are inherently linked to megakaryocytic activity, can induce fibrosis by (i) skewing the activity of MSCs towards fibroblastic and osteoblastic genesis and (ii) increasing the deposition of collagen [55,84,85]. Furthermore, evidence from in vitro experiments on PMF models portrayed CXCL4 as a major artificer of BM fibrosis, being able to (i) upregulate pro-fibrotic pathways in megakaryocytes, (ii) induce glioma-associated oncogene homolog 1 (Gli1)+ MSC migration and differentiation into myofibroblasts and (iii) amplify JAK/STAT activation in both megakaryocytes and MSCs [86]. Further growth factors, such as PDGF and VEGF, subvert the physiological BM niche balance by stimulating myofibroblasts and promoting the maturation and migration of megakaryocytes [82].

In addition to clonally related cell types (i.e., HSCs, megakaryocytes and monocytes), specific subgroups of non-clonal MSCs have emerged as the foremost cellular drivers of inflammation and BM fibrosis, including Gli1+, leptin receptor (LepR)+ and nestin+ MSCs [87]. In particular, Gli1+ and LepR+ MSCs have been identified as the “cellular progenitors” of fibrosis, because of their competence to differentiate into myofibroblasts [88,89]. Of note, unlike monocyte-derived fibrocytes, Gli1+ and LepR+ MSCs express neither the common leukocyte antigen CD45 nor CD34, suggesting two distinct pathways of fibrocyte differentiation [90,91]. Alternatively, under TGF-β stimulation, fibrocytes might lose CD34 and CD45 and start to express smooth muscle actin (a-SMA), thus becoming myofibroblast-like [92,93]. Moreover, several studies showed a markedly altered gene and immunophenotypic expression profile in MPN-associated MSCs, reprogrammed by mutant HSCs in order to overexpress pro-inflammatory/pro-fibrotic factors and ECM components (e.g., glycosaminoglycans, heparan sulfate and chondroitin sulfate) [94]. Finally, a reduction of nestin+ MSCs, which are under the control of the sympathetic nervous system through the neuro-hematopoietic axis, has been described in local neuropathy occurring in the MPN–BM niche [95].

With regard to MPN-associated ECM, noteworthy modifiers comprise matrix metalloproteinases (MMPs) and lysyl oxidases (LOXs). In a study on PMF by Wang and coworkers, MMP3 levels appeared to be inversely correlated with the degree of fibrosis, suggesting that a downregulation of specific MMPs might favor the accumulation of ECM substances [96]. In another study, MMP2 and MMP9 were strongly expressed in patients with MPNs and downregulated after treatment with JAK inhibitors [97]. LOXs, a class of enzymes involved in collagen cross-linking and fibrosis regulation, are physiologically expressed in immature megakaryocytes and downregulated in mature megakaryocytes [98,99,100]. A pathological upregulation of LOXs was detected in MF-associated megakaryocytes, both in murine models and patients, and it has been postulated that the activity of LOXs directly correlates with BM fibrotic changes, as suggested by the different activation patterns observed in PMF (where all LOX members are activated) compared with PV or ET [101].

## 4. The Perfect Storm: Combining Inflammation and Specific Mechanisms of Tumor Immune Escape

**Figure 1:**
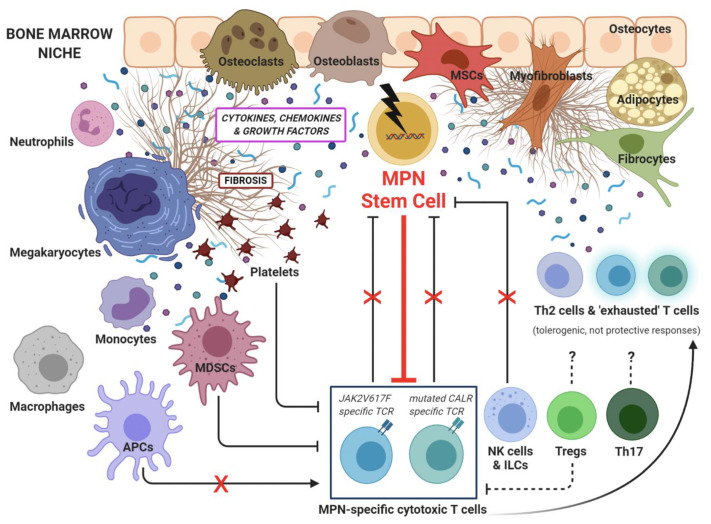
The emerging cancer ecosystem in myeloproliferative neoplasms (MPNs). Several cellular players contribute to the MPN-associated microenvironment, characterized by increased cytokine signaling, fibrosis, inflammation-driven immunosuppression and immune escape. MSCs: mesenchymal stem cells, MDSCs: myeloid-derived suppressor cells, APCs: antigen-presenting cells, TCR: T cell receptor, Th: T helper, NK: natural killer, ILCs: innate lymphoid cells, Tregs: T regulatory cells, ⊥: inhibition, X: block.

A current mainstay of cancer development has become the notion that neoplastic cells may proliferate and emerge as overt disease only when finding successful strategies of immune escape in a permissive tumor microenvironment (TME). In MPNs, the inflammatory TME not only directly promotes the progression of clonal myeloproliferation (see “MPNs as inflammation-driven cancer model” [39], discussed in Section 2), but also provides an important immunosuppressive effect against cytotoxic T cells and other antitumor defenses. In addition, MPN-mutated HSCs themselves have been shown to exert broad pro-inflammatory effects, contributing to a vicious maintenance of the inflammatory TME, as well as to adopt different mechanisms of evasion from T cell immunosurveillance, eventually resulting in uncontrolled clonal escape. A growing set of research works is currently contributing to the depiction of the immunologically disrupted “cancer ecosystem” associated with MPN outgrowth (Figure 1).

To date, robust gene expression studies by Skov et al. [102,103,104], performing whole transcriptional analyses on blood cell populations from MPN patients, disclosed a significant downregulation of human leucocyte antigen-I (HLA-I), HLA-II and other HLA-related genes, as well as of CD40L and FAS, implying a basic impairment of tumor-antigen presentation, as well as of antigen-presenting cell (APC)-mediated costimulatory signaling and T cell cytotoxicity, respectively.

Along with dysfunctional adaptive T cell responses, the arm of innate immunity was also found to be dampened in MPNs: lower levels of circulating natural killer (NK) cells were observed in untreated patients, compared to healthy controls, while a recovery of cytotoxic CD56^bright^ NK cells was associated with long-term IFN-α therapy [105].

Moreover, by focusing on immunologic defects in PMF, Romano and colleagues described quantitative and functional impairments in circulating lymphocyte subsets, such as Th1, Th17, NK and other innate lymphoid cells (ILCs), as well as a reduced capacity of monocytes to differentiate into fully committed dendritic cells (DCs) [106]. In line with this latter finding, in vivo experiments showed that a basic defect of APC functions in DCs (i.e., loss of HLA-II, meaning that DCs fail to properly prime specific T cells) can induce the development of an MPN-like disorder [107]. Intriguingly, in a murine model a combination of deficiencies for both HLA-II and CD4+ T cells completely abrogates the emergence of MPNs, thus suggesting that the presence of “unprimed” CD4+ T lymphocytes (retaining an active pro-inflammatory “effector phenotype”, but without specific cytotoxic abilities against MPN-mutated cells) may be required for the emergence of an “MPN-permissive” TME [107]. Moving from this observation, with regard to a putative role of regulatory T lymphocytes (Tregs) in MPNs, one could first imagine that this suppressive subset may be important in the immunopathogenesis of MPNs, possibly by causing a direct inhibition of specific antitumor responses. However, to date, few immunological studies in MPN patients have shed light on the elusive role of Tregs in this setting, reporting some unexpected and partially discordant data, which may rather suggest that the Treg compartment can be globally impaired (and not expanded) in MPN patients. Indeed, the proportion, phenotype and function of circulating CD4 + CD25 + Foxp3 + T lymphocytes did not significantly differ between untreated MPN patients (6.9%), healthy subjects (6.1%) and MPN patients treated with hydroxyurea (5.8%), while a remarkable expansion of Tregs (13%) was detected in MPN patients undergoing long-term IFN-α therapy [108]. More recently, CD4 + CD127^low^CD25^high^FOXP3 + Tregs were found to be reduced in MPN patients, compared to healthy subjects, but such a decrease became even more profound upon treatment with JAK2 inhibitors (while, on the other hand, Th17 exhibited a slow expansion) [109]. Lastly, in the aforementioned work by Romano et al., Tregs were also numerically contracted and dysfunctional, showing increased cytokine production, but with a globally reduced ability to suppress the proliferation of autologous effector T cells [106]. By taking the data reported to date together, it is emerging that the Treg-mediated suppression of protective T cell responses should not represent a primary requirement for the development of MPNs.

Apart from Tregs, other cell-mediated immunosuppressive strategies have been implied in the immune escape of MPNs from specific T cell defenses. In particular, myeloid-derived suppressor cells (MDSCs) have already shown relevant activities in several hematologic neoplasms, and may represent a crucial link between inflammation and the inhibition of antitumor T cell immunity [110]. Concerning MPNs, CD11b+CD14-CD33+ cells (MDSCs) were significantly more frequent in patients compared to controls, and were associated with higher expression of arginase-1 (ARG1) mRNA and with specific suppressive activity against autologous T lymphocytes [111]. Of note, it has also been hypothesized that MPN-associated clonal thrombocythemia may sustain an intriguing “platelet–cancer loop”, as pathologic platelets could readily suppress specific T cells by means of TGF-β release [112].

Alongside these “indirect” immunosuppressive effects, MPN-mutated cells were also shown to adopt two fundamental mechanisms enabling “direct” suppressive activity against antineoplastic T cells. First, the primary overactivation of JAK/STAT pathways in JAK2V617F + clonal cells (including monocytes, megakaryocytes and platelets) directly induced the overexpression of programmed cell death (PD-1) ligand 1 (PD-L1), thus supporting the idea that MPN cells exploit the PD1/PD-L1 axis to escape specific T cell immunosurveillance [113]. Second, JAK2 mutant cells, by inhibition of ROS-converting enzyme through the upregulation of the PI3K/Akt pathway, are able to produce large amounts of ROS, which are known to negatively affect T cell effector functions [43]. In addition, the detection of clonal T and B lymphocytes (harboring either CALR or JAK2 mutations) suggests that lymphocyte subsets may also have intrinsically defective immune functions [114]. Finally, in the TME of MPNs, paracrine effects of extracellular mutated CALR protein may lead to the functional inhibition of the phagocytosis of cancer cells, further contributing to the escape from antitumor immunity [115].

Altogether, these findings suggest that the immune system is deeply dysregulated in MPNs and that MPNs develop and evolve because of tumor immune evasion. As a matter of fact, by considering that CALR and JAK2 mutations are definitely immunogenic and that specific T cells reactive to these mutations are readily detectable in patients’ peripheral blood (see next section), CALR/JAK2 mutants have to elude such T cell-mediated elimination in order to pathologically expand in the BM. Figure 1 provides a graphical abstract of the MPN-associated TME.

## 5. Novel Mutant Hunters: The Emergence of JAK2/CALR Mutation-Targeted T Cell Immunity

In the past decade, it has been acknowledged that the loss of tumor-specific T cell immunity plays a pivotal role in cancer development, and the restoration of antitumor T cell functions represents a major breakthrough in cancer therapy. Indeed, tumor-specific T lymphocytes are typically suppressed during neoplastic progression, in virtually all cancer types, and are now considered as a fundamental therapeutic target to control tumor outgrowths. Initially, these notions have found basic evidence in the therapeutic effect of “immune system exchange”, provided with bone marrow transplantation, in different settings of hematologic malignancies. Such observations were then confirmed by preclinically and clinically promising results deriving from T cell-based immunotherapeutic approaches, either with allogeneic or autologous cytotoxic T lymphocyte (CTL) infusions, both in hematologic and solid cancer patients. More recently, different groundbreaking immunotherapies have arisen with the development of immune checkpoint inhibitors (ICIs), chimeric antigen receptor T (CAR-T) cells and bispecific T cell engagers (BiTEs) [116,117,118].

Neoantigens are “non-self” epitopes deriving from acquired somatic mutations in tumor cells, and are presented with HLA molecules in a complex that is recognized by T cell receptors (TCRs). Neoantigens, potentially deriving from any protein-coding mutation, fusion protein or cancer-specific splice isoform, are thought to be highly immunogenic, possibly representing optimal targets for cancer immunotherapy [119].

Among hematologic malignancies, neoantigen-specific T cell responses were originally detected in different types of acute and chronic leukemias, as well as in multiple myeloma, often demonstrating significant correlations with disease outcome [120,121,122,123,124,125]. These observations prompted the development of effective immunotherapies with ex vivo expanded specific T lymphocyte infusions (either donor-derived or autologous), which have represented a proof-of-principle of feasibility and efficacy for leukemia-specific CTL treatments [126,127].

During the last five years, ex vivo immunological studies, based on antitumor T cell functional characterizations by peptide-specific ELISpot, flow cytometry and cytotoxicity assays (either directly on peripheral blood samples, or after short-term in vitro expansion), have also been performed for the first time in the setting of MPNs. In particular, Holmström et al. have well disclosed the existence of protective “MPN-specific” T lymphocytes, selectively targeted against transformed cells carrying JAK2V617F and CALR exon 9 mutations [128,129,130]. In more detail, they described that: (i) spontaneous CALRmutant-specific CD4+ T lymphocytes (Th1) are readily detectable in the peripheral blood of many MPN patients; (ii) ex vivo cultured, Th1 cells specific to CALR C-terminus mutations, as well as JAK2V617F-specific CD8+ T lymphocytes, can recognize and eliminate either CALR or JAK2 mutants, respectively, in a mutation-restricted manner. In line with these findings, Bozkus et al. showed that both CD4+ and CD8+ T lymphocytes specific toward CALR C-terminus mutations often emerge in the peripheral blood of patients with CALR-mutated MPNs, but the expression of exhaustion markers (i.e., PD-1 or CTLA-4) reveals their functional impairment, which can be reverted with anti-PD-1 treatment, both in vitro and in patients [131]. Additional longitudinal studies on large MPN patient cohorts are now warranted to provide a complete immunological characterization of circulating MPN-specific T cells (i.e., both CD4+ and CD8+ memory T cell immunoprofiling for the production of different cytokines and functional subsets). In particular, it remains to be assessed whether these mutant-specific T cell responses may be significantly correlated with the disease course, and may represent a valuable prognostic factor in the clinical monitoring of MPN patients. Moreover, MPN-specific T lymphocytes should also be investigated in the BM, which may reveal novel immunobiological features. Interestingly, some surveys indicate that specific memory CD4+ and CD8+ T cell responses, towards either JAK2V617F-positive or CALR C-terminus-mutated HSCs, are detectable not only in MPN patients, but also in healthy subjects [128,131,132]. These findings suggest that some healthy individuals could be specifically protected from the expansion of a transformed myeloproliferative clone, by means of effective T cell immune surveillance. On the other hand, such specific T cells appeared to be lost or weakened in patients with MPNs, further suggesting that a mechanism of immune escape of mutated HSCs from specific T cell patrols should be pivotal in the immunopathogenesis and clinical emergence of MPNs.

Alongside MPN-specific immunity, protective T cell responses directed towards PD-L1 and ARG1 were revealed in about two thirds of MPN cases, showing higher frequencies of these antitumor T lymphocytes in patients with non-advanced disease, compared to weaker magnitudes observed in advanced MPN patients [133,134]. To date, other common tumor antigens (such as WT-1 and PRAME) have not been surveyed yet as targets of spontaneous T cell immunity in MPN patients. Furthermore, a wide in vitro and in silico screening for potential MPN-specific neoantigens recently identified a total of 35 unique putative neoepitopes, deriving from abnormal CALR and MPL splicing variants, due to spliceosome defects, in MPN patients with SF3B1 mutations [135].

In conclusion, the experimental studies reported to date have found that both JAK2V617F mutant cells and different CALR-mutated clones (bearing either type 1 or type 2 frameshift mutations, resulting in “non-self” C-terminal sequences of calreticulin protein) ultimately generate a few distinct highly immunogenic peptides, broadly shared by the majority of MPN patients, thus representing optimal neoantigens which directly provide the opportunity to develop mutant-specific adoptive CTL therapies and vaccination strategies [128,129,130].

## 6. Looking for the Achilles’ Heel in MPNs: Innovative Targeted Therapies

MPN patients experience considerable symptom burden and shortened survival due to thrombohemorrhagic events or progression either to MF or acute myeloid leukemia (AML). In ET and PV, the main goal of treatment is the prevention of the thrombotic complications, while the management strategies for PMF aim at relieving the symptom burden, improving cytopenias, decreasing red blood cell transfusion requirements and modifying the disease course. The only curative treatment option for MPNs is allogeneic hematopoietic stem cell transplantation (allo-HSCT), which may be associated with high treatment-related mortality [136].

In 2011, the FDA approval of ruxolitinib (the first-in-class JAK1/JAK2 inhibitor) for the treatment of MF ushered in the era of JAK inhibitors (JAKi) [137]. Since the oncogenic activity of the CALR and MPL mutations also rely on the JAK-STAT axis, JAKi are effective in patients with wild-type JAK2 MPNs as well. The pharmacologic inhibition of the overactive JAK-STAT signaling provides appreciable clinical benefits in terms of symptom improvement, spleen volume reduction (SVR) and quality of life. Notwithstanding, along with the evident benefits, several limitations of JAKi have become apparent over the years, as JAKi (i) do not improve, or even worsen, cytopenias in most patients, (ii) have relatively modest effects on BM fibrosis, mutation allele burden and survival, (iii) do not prevent progression to AML and (iv) are subject to the development of clinical resistance [138,139]. Thus, it could be argued that JAKi act more as anti-inflammatory rather than definitely disease-modifying agents. Furthermore, 10–15% of MF patients are ineligible for the currently approved JAKi because of severe thrombocytopenia, and significant rates of discontinuation (because of either disease progression/loss of response or toxicities) are registered for ruxolitinib [140,141]. Despite these limits, JAKi form the backbone of MF therapy, and new JAKi are in phase III clinical trials [142]. Finally, there is growing interest in exploring combinations of JAKi with different agents.

### 6.1. Targeting the Microenvironment

The pharmacological blockade of the aberrant “cross-talk” between clonal hematopoiesis and the TME is conceived to be an attractive complementary approach, as this pathological network functionally contributes to disease progression and drug resistance. Various agents exploiting the interconnected pathological pathways in MPNs are in early-phase clinical development (Table 1). Nearly all of them are focused on the care of patients with MF (either primary or post-ET/PV) who failed treatment with ruxolitinib. This second-line setting, characterized by poor outcomes (median survival of 14–18 months) and the absence of effective therapeutic options, represents, indeed, an unmet clinical need [141,143,144].

Megakaryocytes are major cellular drivers of BM fibrosis [145,146,147], and can therefore be considered potential candidates for targeted therapies. Three approaches modulating megakaryocyte maturation have shown clinical efficacy to date: anagrelide, alisertib and bomedemstat. Anagrelide is an inhibitor of megakaryocyte maturation and is currently used as a second-line agent in patients with ET [148,149]. The function of Aurora A kinase (AURKA) is required for the correct duplication and separation of the centrosomes during prophase of mitosis [150]. Targeting AURKA with a specific inhibitor (alisertib) promotes the polyploidization, differentiation and apoptosis of mutant megakaryocytes, reduces the secretion of TGF-β and improves BM fibrosis [151,152]. In a phase I study of higher-risk MF patients, treatment with alisertib led to SVR in 29%, transfusion independence (TI) in 8% and ≥50% symptom improvement in 23% of patients [153]. Bomedemstat inhibits lysine-specific histone demethylase 1 (LSD1 or KDM1A), whose function is fundamental for platelet formation [154]. In January 2020, the FDA granted fast track designation to bomedemstat for use in ET (NCT04254978). In murine models of MF, treatment with bomedemstat allowed a reduction of inflammation, spleen size and fibrosis, as well as improved survival [155]. A phase II clinical trial (NCT03136185) exploring the efficacy of bomedemstat in MF patients is underway [156].

Gli1 + MSCs, which are pivotal in the development of fibrosis, constitute a further potentially targetable cell population. In preclinical in vitro models, the use of GANT61, an inhibitor of the Gli1 transcription factor (the nuclear mediator of the Hedgehog pathway), enabled an improvement of BM fibrosis [88].

The antifibrotic compound PRM-151 is a recombinant form of pentraxin-2, an endogenous human protein that modulates the differentiation of monocytes into fibrocytes, thereby preventing and reverting fibrosis [157].

The TGF-β superfamily, including activins, growth differentiation factors and bone morphogenetic proteins, plays a major role during signaling in the BM niche, being able to promote oxidative stress, myeloproliferation and fibrosis [55,85,158]. Sotatercept and luspatercept are erythroid maturation agents that work as activin receptor ligand traps of IIA and IIB, respectively [159,160,161,162]. Luspatercept is currently approved for the treatment of anemia in low-risk myelodysplastic syndromes (MDSs) with ring sideroblasts [163]. In a phase II study of MF patients with anemia, an erythroid response was observed in six of 17 patients (35%) treated with sotatercept alone, and in one of eight patients (12.5%) treated with sotatercept in combination with ruxolitinib [164]. Similarly, in a recent study of luspatercept in anemic patients with MF, 10% and 32% of patients reached TI in the monotherapy arm and combination arm, respectively, while a sustained hemoglobin increase was registered in 10% and 21% of transfusion-independent patients, respectively [165]. The most common treatment-related adverse events (TRAEs) in both studies were hypertension, diarrhea and bone pain. AVID200 is a ligand trap that selectively inhibits the putatively fibrogenic isoforms of TGF-β (TGF-β1 and TGF-β3), whilst sparing the TGF-β2 isoform, which promotes hematopoiesis [166]. The efficacy of AVID200 in MF is under evaluation in a multicenter phase I/Ib study (NCT03895112). Further strategies to target this pathway encompass monoclonal antibodies against TGF-β [167] and kinase inhibitors against the TGF-β receptor I kinase [168].

Moreover, the therapeutic modulation of the neuro-hematopoietic axis has recently been explored in MPNs [169]. In a phase II trial, 39 patients with a JAK2V617F-mutated MPN were treated for 24 weeks with an oral β-3 adrenergic agonist (mirabegron). Although the primary end point (>50% reduction in JAK2V617F allelic burden) was not reached for any of the patients, BM evaluation (available for 20 out of 39 patients) showed an increase of the nestin+ MSCs, a slight decrease in the reticulin fibers and a trend towards a reduction of megakaryocyte clusters [170].

Since the progressive deposition of ECM proteins is characteristic of MF [171], a further therapeutic approach is to normalize the composition of the ECM. As described above, LOXs play a key role in this process by cross-linking collagens and elastins through the deamination of lysins and hydroxylysins [98]. However, simtuzumab, a humanized monoclonal antibody targeting lysyl oxidase-like 2 (LOXL2), failed to meet expectations from in vitro studies [172,173], when tested in a phase II clinical trial of MF patients [174].

Despite some encouraging results, further development of these compounds is uncertain, as their spectrum of activity is generally restricted to a single (such as antifibrotic or erythroid-maturating) effect, with low global disease-modifying potential when employed as monotherapy. Relevant to this, it should be noted that the exact relationship between BM fibrosis and clinical outcomes in MF is not fully defined [175]. Therefore, whether a decrease in BM fibrosis necessarily correlates with a clinical benefit in terms of symptomatic improvement, SVR and prolonged survival is still a matter of debate. However, it seems advisable that these new drugs could fruitfully be exploited as “add-ons” with JAKi in a multitargeted approach.

### 6.2. Targeting CD123

IL-3 is part of a family of cytokines involved in the regulation of the growth, differentiation and migration of HSCs. The IL-3 receptor belongs to the type I cytokine receptor family and is a heterodimer with a unique alpha chain (IL-3Rα or CD123) paired with CD131 (common beta chain, βc) [176]. The binding of IL-3 to CD123 is followed by the recruitment of CD131, assembly of the receptor complex and engagement of downstream signaling through JAK2 [177]. Excessive signaling due to the overexpression of either IL-3 or its receptors has been associated with pathological phenomena such as inflammatory diseases and hematological malignancies [178]. CD123 is a marker of blastic plasmacytoid dendritic cell neoplasm (BPDCN), but it is also overexpressed in other cancers, including AML, MDS, systemic mastocytosis, acute lymphoblastic leukemia, Hodgkin lymphoma and hairy cell leukemia [178,179,180]. In AML, CD123 was found to be highly expressed on both leukemic stem cells (LSCs) and more differentiated leukemic blasts, whereas the normal CD34+ counterpart lacks this surface marker [181]. Moreover, CD34+ CD38− CD123+ cells are able to engraft and recapitulate the development of leukemia in immunodeficient mice, as demonstrated by Jordan and colleagues [181]. The overexpression of CD123 on AML cells is associated with constitutive phosphorylation of STAT5, increased cell-cycle activity, reduced apoptosis, higher burden of disease and negative prognosis [182]. In MF, 1–2% of circulating cells are CD123+, 30–50% of which co-express CD13, CD16 or CD11b, representing monocytes and immature myeloid cells [183]. These findings put forward the concept that CD123 can be exploited both as a biomarker and a therapeutic target, since CD123 blockade may enable an antileukemic effect, whilst preserving normal hematopoietic cells [184]. SL401 (tagraxofusp), a recombinant protein composed of a truncated diphtheria toxin fused to IL-3, was approved by the FDA in 2018 for the use in pediatric and adult patients with BPDCN [185], and has also exhibited some clinical efficacy in MDS and AML [186,187]. The shared phylogeny of plasmacytoid DCs and monocytes, alongside the poor outcomes observed in MF patients with peripheral monocytosis, also inspired the clinical evaluation of SL401 in the settings of relapsed/refractory MF and chronic myelomonocytic leukemia (CMML). An ongoing clinical trial (Table 2) aims at establishing the highest tolerated dose (stage 1) and assessing the safety and efficacy (stage 2) of SL401 in patients with MF or high-risk CMML. According to the interim data published in 2019, 27 patients with MF have been treated with SL401, including 14 patients who had previously received ≥3 lines of therapy. SVR was observed in 53% of evaluable patients with baseline splenomegaly, while the symptom response rate was 45%. The most frequent TRAEs included headache, hypoalbuminemia, increased levels of alanine aminotransferase, thrombocytopenia and anemia; these hematological toxicities also represented the most common ≥grade 3 TRAEs [188].

### 6.3. Checkpoint Inhibitors

ICIs, a class of immunomodulatory monoclonal antibodies targeting the PD1/PDL-1 axis, CTLA-4 and other suppressive T cell signaling pathways, have been demonstrated to induce functional recovery of antineoplastic T cell immunity, and this was associated with impressive therapeutic responses in a variety of tumor patients at different stages of disease [116]. The pharmacological retrieval of protective T cell responses could also represent an attractive strategy in the MPN setting, either alone or as an immunogenic boost for other immunotherapies (such as vaccines or adoptive T cell therapies). However, to date, none of the ICIs has been approved for use in myeloid neoplasms. The recognition of an oncogene-driven immune escape of JAK2V617F mutant cells via the JAK/STAT/PD-L1 axis provides the biological rationale for the use of ICIs in MPNs [113]. On the basis of preclinical data, clinical trials (Table 2) were planned in order to address the safety and efficacy of ICIs in the setting of MPNs. The final results have not been published for any of the studies, and two out of four trials were prematurely terminated. An ongoing clinical trial (NCT03065400) is evaluating the effectiveness of pembrolizumab, administered at a dose of 200 mg via intravenous infusion, every 3 weeks for six planned cycles, in patients with MPNs; in case of clinical improvement after six cycles, patients will continue to receive the drug until evidence of disease progression, unacceptable toxicity or patient or physician decision, for a maximum of 2 years. A further phase I clinical trial (NCT01822509) aims at assessing the safety and the best dose of ipilimumab or nivolumab in patients with diverse hematological malignancies who relapsed after allo-HSCT.

### 6.4. Vaccination

Cancer neoantigens, arising from acquired somatic mutations, theoretically represent optimal immunotherapeutic targets, as they are uniquely expressed by the neoplastic cells and are able to elicit neoantigen-specific T cells, which do not undergo negative selection during the development of central tolerance. Therefore, enhancing neoantigen-specific immune responses through cancer vaccination or adoptive T cell therapies actually constitutes a fascinating approach. Nevertheless, the clinical development of cancer vaccines has been generally hindered by the high genetic heterogeneity of most malignancies, which carry multiple somatic mutations potentially resulting in a myriad of different neoantigens. Such a complex scenario would require a personalized (i.e., patient-specific) vaccine design, based on the sequencing of the mutanome of each patient. Cancer-induced immune impairment represents an additional critical hurdle, as it might impair the clinical efficacy of protein/peptide vaccination, possibly accounting for the disappointing outcomes reported to date, especially in advanced-stage neoplasms [189].

Akin to solid tumors, MPNs express high levels of immunosuppressive proteins, such as PD-L1 and ARG1, as described above, whereas a highly variegated mutational spectrum is not characteristically observed in this group of neoplasms, since 80–90% of patients harbor either JAK2V617F or CALR mutations, which are thought to be relevant sources of shared neoantigens. Importantly, Holmström et al. identified spontaneous T cell responses against both JAK2V617F and CALR exon 9-derived epitopes, paving the way for targeted immunotherapeutic strategies in the setting of MPNs [128,129,130]. The same authors also documented the presence of specific T cell responses against PD-L1 and ARG1 in most patients with MPNs; more importantly, non-advanced cases exhibited stronger and more frequent immune responses compared to patients with advanced MPNs, highlighting the fundamental role of immune evasion in the progression of disease [134]. In line with this notion, patients with PV and ET, rather than those with PMF (whose immune responses are quantitatively and functionally impaired), are supposed to benefit the most from a therapeutic vaccination with either mutant- or PD-L1/ARG1-derived epitopes. On the basis of these findings, two clinical trials have been launched, in order to evaluate the safety and immunogenicity of peptide vaccines, targeting mutated CALR and PD-L1/ARG1, respectively (Table 2).

## 7. Conclusions

**Figure 2:**
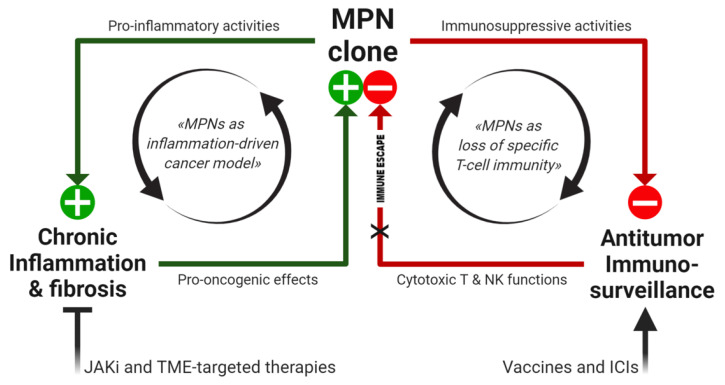
Working model of MPN immunopathogenesis and related immunotherapeutic strategies. In the MPN-associated BM niche, relevant immunological dynamics are sustained by the mutant clone, creating positive feedback loops (black arrows), which can directly promote tumor progression (through chronic inflammation, inducing genomic instability and ROS-dependent mutagenesis) as well as support the permissive microenvironment (by loss of MPN-specific protective immunity). Both immunopathogenetic features can be targeted by novel therapeutic approaches, aiming to inhibit JAK2 constitutive signaling, modulate the TME and restore antitumor immunosurveillance. JAKi: JAK inhibitors, TME: tumor microenvironment, ICIs: immune checkpoint inhibitors, ⊥: inhibition, X: block.

The inflammatory microenvironment and loss of specific T cell immunity represent the emerging immunopathogenetic features of MPNs, which rely on constitutive activation of the JAK/STAT pathway, induced by recurrent acquired mutations. Beyond JAKi, innovative therapeutic strategies addressing MPN immunological signatures are now in the pipeline (Figure 2).

At present, by considering (a) growing evidence on the protective role of MPN-specific T lymphocytes, and (b) previous experiences describing successful clinical translation of ex vivo investigations on tumor-specific T cell immunity, into the development of either adoptive or active T cell therapies, it seems time to try exploiting the antitumor potential of MPN-specific T lymphocytes in the therapeutic management of MPN patients. In particular, novel “T cell-based” immunotherapies may serve to hunt and eliminate residual mutated HSCs in patients with an ongoing response to “molecular” treatments against the JAK/STAT pathway.

## Figures and Tables

**Table 1:** Selected drugs targeting the MPN microenvironment.

Drug	Target (Mechanism)	Condition(s)	Trial (Phase)	Reference	Results/Comments
**PRM-151**	Recombinant human pentraxin-2	MF	NCT01981850 (Phase II)	Verstovsek et al., 2019 [157]	↓fibrosis, ↓transfusions, only modest SI
**Fresolimumab (GC1008)**	TGF-β (MoAb)	MF	NCT01291784 (Phase I)	Mascarenhas et al., 2014 [167]	Only 3 patients enrolled; early termination
**Sotatercept** **(ACE-011)**	TGF-β (ligand trap)	MF with anemia	NCT01712308 (Phase II)	Bose et al., 2016 [164]	±ruxolitinibEndpoint: ↑Hb ORR (monotherapy) = 35%
**Luspatercept** **(ACE-536)**	TGF-β (ligand trap)	MF with anemia	NCT03194542 (Phase II)	Gerds et al., 2019 [165]	±ruxolitinibEndpoint: ↑Hb ORR (monotherapy) = 20%
**AVID200**	TGF-β1/β3 (ligand trap)	MF	NCT03895112 (Phase I/Ib)		Still recruiting; no results published yet
**Galunisertib (LY2157299)**	ALK5 (kinase inhibitor)	MF	Preclinical	Yue et al., 2017 [168]	Murine models only: ↓fibrosis
**Alisertib** **(MLN8237)**	AURKA (kinase inhibitor)	MF (or AMKL)	NCT02530619 (Phase I)	Gangat et al., 2019 [153]	SVR = 29%, TI = 8%, SI = 23%, ↓fibrosis
**Bomedemstat** **(IMG-7289)**	LSD1 (small molecule inhibitor)	MF	NCT03136185 (Phase II)	Pettit et al., 2019 [156]	Still recruiting; interim results: SI, slight SVR in a subset of patients
**Mirabegron**	β-3 adrenergic agonist	JAK2V617F+ MPNs	NCT02311569 (Phase II)	Drexler et al., 2019 [170]	↑nestin + MSCs, mild ↓fibrosis, ↓MK clusters, ↔ JAK2V617F allele burden
**Simtuzumab** **(GS-6624)**	LOXL2 (MoAb)	MF	NCT01369498 (Phase II)	Verstovsek et al., 2017 [174]	±ruxolitinib ↓Fibrosis in 36.7%; limited overall efficacy
**GANT61**	Gli1/Hedgehog (small molecule inhibitor)	MF	Preclinical	Schneider et al., 2017 [88]	Murine and human in vitro models: ↓fibrosis, ↓myofibroblastic phenotype

MF: primary or post-essential thrombocythemia/polycythemia vera myelofibrosis; MoAb: monoclonal antibody; SVR: spleen volume reduction; SI: symptom improvement; Hb: hemoglobin; ORR: overall response rate; TI: transfusion independence; MK: megakaryocyte; AMKL: acute megakaryoblastic leukemia; ALK5: TGF-β receptor I kinase; AURKA: Aurora kinase A; LSD1: lysine-specific histone demethylase 1; LOXL2: lysyl oxidase-like 2; ↓: decrease; ↑: increase; ↔: no effects; ±: with or without; %: percentage of patients.

**Table 2:** Selected clinical trials investigating anti-CD123, immune checkpoint inhibitors (ICIs) and tumor vaccination in MPNs.

Drug	Target (Mechanism)	Condition(s)	Trial (Phase)	Reference	Results/Comments
**Tagraxofusp** **(SL-401)**	CD123 (MoAb)	MF (or CMML)	NCT02268253 (Phase I/II)	Pemmaraju et al., 2019 [188]	Still recruiting; SVR = 53%, SI = 45%
**Nivolumab**	PD-1 (MoAb)	MF	NCT02421354 (Phase II)	-	Terminated early for lack of efficacy
**Pembrolizumab**	PD-1 (MoAb)	PV, MF	NCT03065400 (Phase II)	Cimen Bozkus et al., 2019 [131]	Ongoing; ↑reactivity to CALR mutant epitopes in vivo and in vitro
**Durvalumab**	PD-L1 (MoAb)	MF	NCT02871323 (Phase I)		Withdrawn before patients’ enrolment
Ipilimumab *	CTLA-4 (MoAb)	MPNs (among other conditions)	NCT01822509 (Phase I/Ib)		Ongoing; aims: assessment of AEs and best dose of ipilimumab or nivolumab
**CALRLong36 peptide (exon 9 mut) vaccine**	Mutated CALR (vaccine)	CALR+ PMF and ET	NCT03566446 (Phase I)		Aims: assessment of AEs and T cell cytokine release against the target antigen
**PD-L1Long (19–27)** **ArgLong2 (169–206) vaccine**	PD-L1 and ARG1 (vaccine)	ET, PV	NCT04051307 (Phase I/II)		Expected effects: ↑specific T cell responses, ↑killing of mutant cells, ↓ARG1, ↓PD-L1

MF: primary or post-essential thrombocythemia/polycythemia vera myelofibrosis; MoAb: monoclonal antibody; CTLA-4: cytotoxic T lymphocyte antigen-4; AEs: adverse events; SVR: spleen volume reduction; SI: symptom improvement; CMML: chronic myelomonocytic leukemia; ↓: decrease; ↑: increase; %: percentage of patients. * Ipilimumab or nivolumab in patients with various hematological neoplasms (including MPNs) relapsed after allogeneic hematopoietic stem cell transplantation.
